# Outlier Detection using Projection Quantile Regression for Mass Spectrometry Data with Low Replication

**DOI:** 10.1186/1756-0500-5-236

**Published:** 2012-05-15

**Authors:** Soo-Heang Eo, Daewoo Pak, Jeea Choi, HyungJun Cho

**Affiliations:** 1 Department of Statistics, Korea University, Seoul, Korea

## Abstract

**Background:**

Mass spectrometry (MS) data are often generated from various biological or chemical experiments and there may exist outlying observations, which are extreme due to technical reasons. The determination of outlying observations is important in the analysis of replicated MS data because elaborate pre-processing is essential for successful analysis with reliable results and manual outlier detection as one of pre-processing steps is time-consuming. The heterogeneity of variability and low replication are often obstacles to successful analysis, including outlier detection. Existing approaches, which assume constant variability, can generate many false positives (outliers) and/or false negatives (non-outliers). Thus, a more powerful and accurate approach is needed to account for the heterogeneity of variability and low replication.

**Findings:**

We proposed an outlier detection algorithm using projection and quantile regression in MS data from multiple experiments. The performance of the algorithm and program was demonstrated by using both simulated and real-life data. The projection approach with linear, nonlinear, or nonparametric quantile regression was appropriate in heterogeneous high-throughput data with low replication.

**Conclusion:**

Various quantile regression approaches combined with projection were proposed for detecting outliers. The choice among linear, nonlinear, and nonparametric regressions is dependent on the degree of heterogeneity of the data. The proposed approach was illustrated with MS data with two or more replicates.

## Findings

### Background

Mass spectrometry (MS) data are often generated from various biological or chemical experiments. Such vast data is usually analyzed automatically in a computer process consisting of pre-processing, significance test, classification, and clustering. Elaborate pre-processing is essential for successful analysis with reliable results. One pre-processing step is required to detect outliers, which which are extreme due to technical reasons. The plausible outlying observations detected can be examined carefully, and then corrected or eliminated if necessary. However, as the manual examination of all observations for outlier detection is time-consuming, plausible outlying observations must be detected automatically.

Identification of statistical outliers is the subject of some controversy in statistics [1]. Several outlier detection algorithms have been proposed for univariate data, including Grubbs’ test [2] and Dixon’s Q test [3]. These tests were designed to analyze data under the normality assumption, so that they may produce unreliable outcomes in the case of few replicates. Furthermore, they are not applicable for duplicated samples. Another naive approach to detect outliers statistically constructs lower and upper fences of differences between two samples, *Q*_1_ - 1.5 *IQR* and *Q*_3_ + 1.5 *IQR*, where *Q*_1_ is the lower 25% quantile, *Q*_3_ is the upper 25% quantile, and *IQR* = *Q*_3_ - *Q*_1_. They are claimed to be outliers if they are smaller than the lower fence or larger than the upper fence. However, this may generate a spurious result because variability is heterogeneous in high-throughput data even generated from MS experiments.

**Figure 1:**
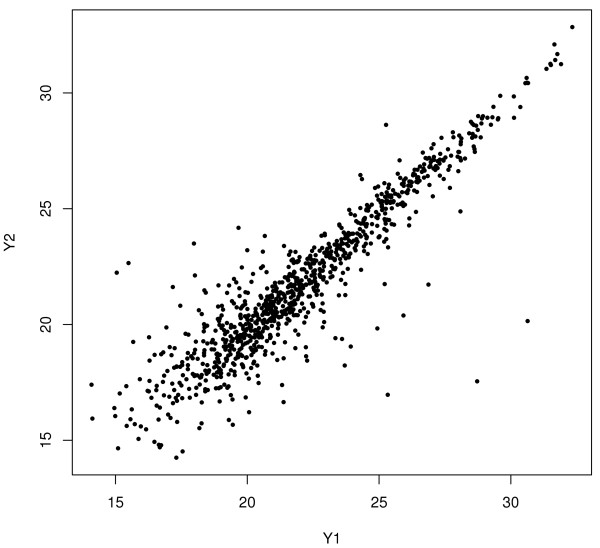
**Scatter plot of duplicate samples.** File: Scatter.pdf - Scatter plot of duplicate samples after log2 transformation from mass spectrometry proteomics data.

Figure 1 shows the log-scale scatter plot of the technically duplicated samples under the same biological condition from a MS experiment. The variability differs according to the intensity levels in the plot, so that the naive outlier detection method, ignoring the heterogeneity of variability, may often miss true outliers at high levels and select false outliers at low levels. If a number of technical replicates for each peptide under the same biological condition can be obtained in MS experiments, the examination of outliers can be conducted for each peptide. However, a small number of replicates is usually conducted for MS experiments due to the high cost of experiments and the limited supply of biological samples.

Cho et al. [4] proposed a more elaborate approach for detecting outliers with low false positive and negative rates in MS data to solve the problem when the number of technical replicates is two. The algorithm was developed by utilizing quantile regression for duplicate MS experiments. The R package (called *OutlierD*) that was also developed can only be used for *duplicate* experiments. Therefore, we here propose a new outlier detection algorithm for *multiple* high-throughput experiments, particularly those with few, but more than two replicates.

### Classical Approaches

Suppose that there are *n* replicated samples and *p* peptides in MS data. Then let *x*_*ij*_ be the *i*th replicated sample from experiments under the same biological or experimental condition, where *i *= 1,…,*n* and *j *= 1,…,*p*. For convenience, let $y_{\text{ij}}=\underset{2}{\text{log}}(x_{\text{ij}})$. Typically, *n* is small and *p* is very large in high-throughput data, *i.e.*, *p *>>* n*. In addition, let *y*_(1)*j*_ ≤* y*_(2)*j*_≤⋯≤* y*_(*n*)*j*_ be ordered samples for peptide *j*, where $y_{(1)j}=\underset{1\leq i\leq n}{\text{min}}y_{\text{ij}}$ and $y_{(n)j}=\underset{1\leq i\leq n}{\text{max}}y_{\text{ij}}$, the smallest and the largest observations, respectively.

Outliers are often detected by the classical approaches such as Dixon’s Range Test and Grubbs test. Dixon’s Range Test, also known as Dixon’s Q-test [3], utilizes order statistics as follows.

(1) $$Q_{j}=\frac{\left(y_{(2)j}-y_{(1)j}\right)}{\left(y_{(n)j}-y_{(1)j}\right)}\;\;\;\text{or}\;\;\;\frac{\left(y_{(n)j}-y_{(n-1)j}\right)}{\left(y_{(n)j}-y_{(1)j}\right)}.$$

The denominator is the difference between the largest and smallest observations and the numerator is the difference between the smallest two values or the largest two values. If the test statistic *Q*_*j*_ is smaller than the critical value given by Rorabacher [5], peptide *j* is flagged as an outlier. If *n *= 2, the statistic is always 1; thus, this test is applicable for *n *≥ 3.

Grubbs’ test [2,6] also utilizes order statistics and its test statistic is defined as follows.

(2) $$T_{\text{nj}}=\frac{\left(y_{(n)j}-{\bar{y}}_{\cdot j}\right)}{s_{j}}\;\;\;\text{and}\;\;\;T_{1j}=\frac{\left({\bar{y}}_{\cdot j}-y_{(1)j}\right)}{s_{j}},$$

where ${\bar{y}}_{\cdot j}$ is the sample mean and *s*_*j*_ the standard deviation for peptide *j*. The denominator is the standard deviation and the numerator is the difference between the smallest (or largest) value and the sample mean. If *T*_*nj*_ or *T*_1*j*_ is smaller than the critical value, peptide *j* is flagged as an outlier. If n = 2, the statistic is always $1/\sqrt{2}$; thus, this test is also applicable for *n *≥ 3.

### Proposed Methods

In duplicated experiments (*n* = 2), two observed values, *x*_1*j*_ and *x*_2*j*_ for each *j*, should be theoretically identical, but are not identical in practice due to their variability. Even though they are not identical, they should not differ substantially. The tolerance of the difference between the two observed values from the same condition is not constant because their variability is heterogeneous. The variability of high-throughput data depends on intensity levels.

Cho et al. [4] proposed the construction of lower and upper fences using quantile regression in an MA plot with *M* and *A* values in vertical and horizontal axes, respectively, where *M*_*j*_ is the difference between replicated samples for *j* and *A*_*j*_ is the average, *i.e.*$M_{j}=y_{1j}-y_{2j}=\underset{2}{\text{log}}(x_{1j}/x_{2j})$ and $A_{j}=(y_{1j}+y_{2j})/2=(1/2)\underset{2}{\text{log}}(x_{1j}x_{2j})$ to detect the outliers accounting for the heterogeneity of variability.

**Figure 2:**
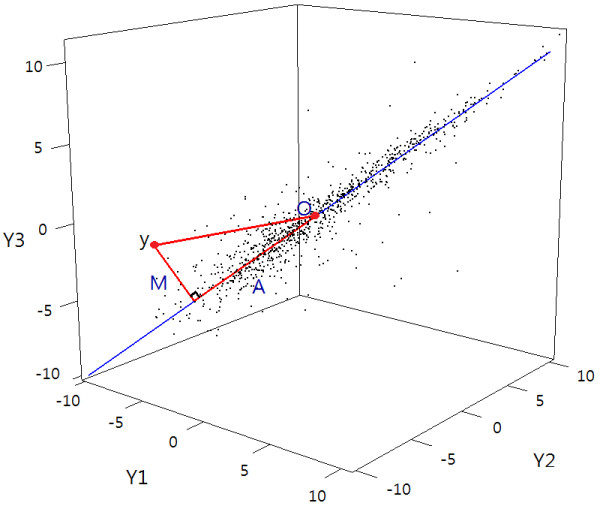
**Outlier detection using projection quantile regression.** File: MA.pdf - Outlier detection using projection quantile regression for mass spectrometry data. The dotted lines represent *Q*_3_(*A*) and the solid lines represent upper fences classifying outliers and non-outliers.

In multiple experiments (*n *≥ 2), it is natural to investigate outliers based on all observed values in a high-dimensional space. An outlier will be a very large distance from the center of the distribution of a peptide. The cutoffs of distances for classification of outliers depend on the degree of variability from the center. The degree of variability is dependent on intensity levels and the center can be defined as the 45° line from the origin. More flexibly, the center can be obtained by principal component analysis (PCA), as seen in Figure 2. The first principal component (PC) becomes the center of each intensity level, *i.e.*, a new axis for intensity levels. The experiments are replicated under the same biological and technical condition; hence, most variation can be explained by the first PC. It implies that it is enough to use the first PC practically. An outlier will have a large distance from its projection. Following the notations for applying quantile regression, we can define the distance of peptide *j* to the projection as *M*_*j*_ and the length of the projection on the new axis as *A*_*j*_. Then the first and third quantiles can be obtained by applying quantile regression on an MA plot with *M* and *A* in the vertical and horizontal axes, repectively; hence, the upper and lower fences can be constructed to classify the outliers.

Describing this projection approach in more detail, we first subtract the sample mean of each sample from each observation to shift the sample mean to the origin because the PC go through the sample means. The first PC vector **v** can be found on the new sample space from $y_{1}^{*},\ldots ,y_{n}^{*}$ and the projection of each peptide on the vector **v **can be obtained. Then, we can calculate the length of the projection, $|{y_{j}^{*}}^{\prime }v|/\sqrt{v^{\prime }v}$, and the length of the difference between a vector of peptide *j* and the projection, $\left|y_{j^{*}}-({y_{j}^{*}}^{\prime }v/v^{\prime }v)v\right|$. The length of the projection is multiplied by the sign of ${y_{j}^{*}}^{\prime }v$ to distinguish the positive and negative directions. The signed length of the project and the length of the difference are defined as *A*_*j*_ and *M*_*j*_ of peptide *j*, respectively. Outlying peptides will have unduly large *M* values. Judging whether it is undue or not depends on *A*_*j*_ because the variability of *M* values is heterogeneous. Like *OutlierD*, we obtain first and third quantiles, *Q*_1_ and *Q*_1_, depending on intensity levels, and then construct the upper and lower fences to classify outliers from normal observations. Quantile regression [7] is utilized on an MA plot to obtain the first and third quantile estimates, *Q*_1_(*A*) and *Q*_3_(*A*), respectively, depending on the intensity levels *A*. The *q*-quantile *linear* quantile regression with {(*A*_*j*_*M*_*j*_),*j *= 1,…,*p*} is used to find the parameters minimizing

(3) $$\begin{array}{lll} & \underset{\left\{j:M_{j}\geq g(A_{j};\theta_{0},\theta_{1})\right\}}{\sum }q|M_{j}-g(A_{j};\theta_{0},\theta_{1})|\;+\; & \;\\ & \underset{\left\{j:M_{j}<g(A_{j};\theta_{0},\theta_{1})\right\}}{\sum }(1-q)|M_{j}-g(A_{j};\theta_{0},\theta_{1})|\; \end{array}$$

where 0 <* q *< 1, and *g*(*A*_*j*_;*θ*_0_*θ*_1_) =* θ*_0_ + *θ*_1_*A*_*j*_. Using Equation (3), the 0.25 and 0.75 quantile estimates, *Q*_1_(*A*) and *Q*_3_(*A*), are calculated depending on the levels *A*. Then, the lower and upper fences are constructed: *Q*_1_(*A*) - *kIQR*(*A*) and *Q*_3_(*A*) + *kIQR*(*A*), where *IQR*(*A*) = *Q*_3_(*A*) - *Q*_1_(*A*) and *k* is a tuning parameter. We set *k* to 1.5 as the default value in our algorithm and software program because the value is practically often used. A larger *k* value selects fewer peptides, while a smaller *k* selects more outliers. The value can be adjusted empirically according to the magnitude of the variation of the data.

We can obtain more flexible quantile estimates by *nonlinear* and *nonparametric* quantile regression approaches [8]. For nonlinear quantile regression, the asymptotic function [9] can be employed:

(4) $$g(A_{j};\theta_{1},\theta_{2},\theta_{3})=\theta_{1}\{1-\text{exp}[-\text{exp}(\theta_{2})\times (A_{j}-\theta_{3})]\},$$

where *θ*_1_ is the asymptote, *θ*_2_ is the log rate, and *θ*_3_ is the value of *A* at which the response becomes zero. In addition, Self-starting, Frank, Asymptotic with Offset and Copula functions can be employed. For nonparametric quantile regression, we utilize smoothing spline with the total variation regularization for univariate data to our algorithm [10]. A smoothing parameter plays a role in adjusting the degree of smoothness. We set it to 1 as the default, but it can be changed by users. The algorithm using projection can be summarized as follows.

### Proposed Algorithm

1. Shift the sample means $\left({\bar{y}}_{1},\ldots ,{\bar{y}}_{n}\right)$ to the origin (0,…,0), *i.e.*, $y_{\text{ij}}^{*}=y_{\text{ij}}-{\bar{y}}_{i}$.

2. Find the first PC vector **v**using PCA on the space of $y_{1}^{*},\ldots ,y_{n}^{*}$.

3. Obtain the projection of a vector $y_{j}^{*}=(y_{1j}^{*},\ldots ,y_{\text{nj}}^{*})$ of each peptide *j* on **v**, where *j *= 1,…,*p*.

4. Compute the signed length of the projection $A_{j}=\text{sign}({y_{j}^{*}}^{\prime }v)|{y_{j}^{*}}^{\prime }v|/\sqrt{v^{\prime }v}$ and the length of the difference between a vector of peptide *j* and the projection $M_{j}=|y_{j}^{*}-({y_{j}^{*}}^{\prime }v/v^{\prime }v)v|$, where *j *= 1,2,…,*p*.

5. Obtain the first and third quantile values *Q*_1_(*A*) and *Q*_3_(*A*), on an MA plot using a quantile regression approach. Then calculate *IQR*(*A*) =* Q*_3_(*A*)−*Q*_1_(*A*).

6. Construct the lower and upper fences, *LB*(*A*) = *Q*_1_(*A*) - *k IQR*(*A*) and *UB*(*A*) = *Q*_3_(*A*) + *k IQR*(*A*), where *k* is a tuning parameter.

7. Declare peptide *j* as an outlier if it is located above the upper fence or under the lower fence.

This projection approach utilizes all the replicates simultaneously, and a high-dimensional problem reduces to two-dimensional one that can easily be solved. Shifts from biased experiments can be ignored due to the use of PCA.

## Results and discussion

We conducted a simulation study to investigate the performance of the proposed approaches. We also applied it to real-life data with three replicates of liquid chromatography/tandem MS (LC-MS/MS) experiments.

### Simulated data

**Figure 3:**
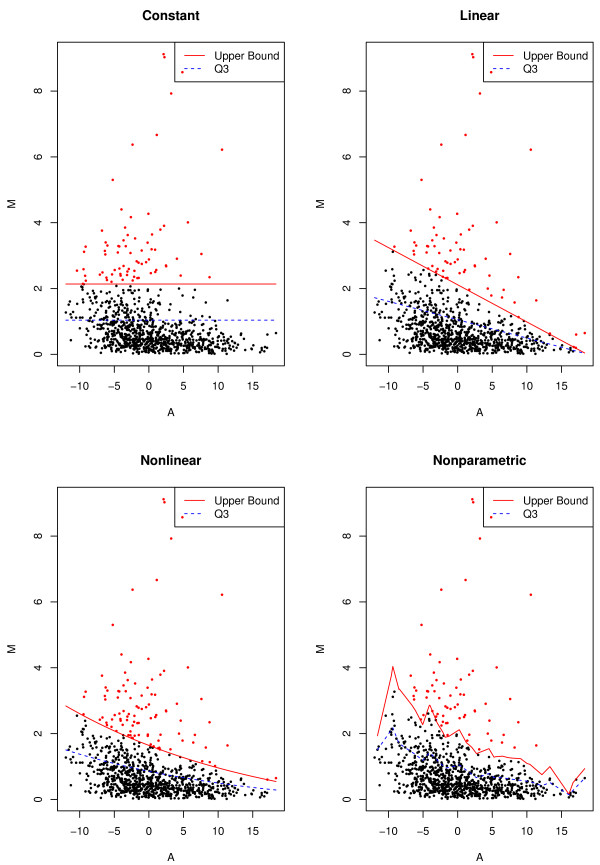
**Relationship between mean and variance for simulated data.** File: Var.pdf - Constant, linear, nonlinear, and nonparametric relationship between *μ *and *σ *to generate the simulated data.

Suppose that there are replicated samples with *p* = 1000 peptides. We considered two or more replicates, *i.e.*, *n *≥ 2. Assimilating reality, we first drew the means *μ*_*j*_ from U(5,35) and computed the variances $\sigma_{j}^{2}$ with the following relationships between the mean *μ* and variance *σ*^2^.

(5) $$\begin{array}{ll} \text{Constant}: & \sigma_{j}=1\\ \text{Linear}: & \sigma_{j}=-(\mu_{j}-5)/10+3\\ \text{Nonlinear}: & \sigma_{j}=\text{exp}(2-\mu_{j}/10)\\ \text{Nonparametric}: & \sigma_{j}=\text{exp}(2-\mu_{j}/10)+(2B_{j}-1)Z_{j} \end{array}$$

where *B*_*j *_∼Bernoulli(1/2) and *Z*_*j *_∼N(1/*μ*_*j*_,0.01). The relationships between the means and the variances are shown in Figure 3. For 950 non-outliers (*j *= 1,…,950), we assumed that $Y_{\text{ij}}∼N(\mu_{j},\sigma_{j}^{2})$ for *i *= 1,…,*n*. For 50 outliers (*j *= 951,…,1000), we assumed that $Y_{\text{ij}}∼N(\mu_{j}^{\prime },\sigma_{j}^{2})$ for one of the samples and $Y_{\text{ij}}∼N(\mu_{j},\sigma_{j}^{2})$ for the other samples, where *μ*_*j *_∼ U(5,35) and $\mu_{j}^{\prime }$=*μ*_*j*_ + (2*B*_*j *_−1)U(1,2) for constant variance and $\mu_{j}^{\prime }$=* μ*_*j*_ + (2*B*_*j *_−1)(120/*μ*_*j*_)U(1,2) for other variances. Thus, an artificial data set for each *n* was generated with 950 non-outliers and 50 outliers. Then, the data were used to check the sensitivities (the probabilities of detecting outliers correctly), specificities (the probabilities of detecting non-outliers correctly), and accuracies (the probabilities of detecting outliers or non-outliers correctly) of the quantile and projection quantile approaches for *n *= 2 and the Dixon test, Grubbs’s test, and projection quantile approaches for *n *= 3,…,8. Constant, linear, nonlinear, and nonparametric quantile regressions were accounted for the quantile and projection quantile approaches. This procedure was repeated 1000 times independently.

**Figure 4:**
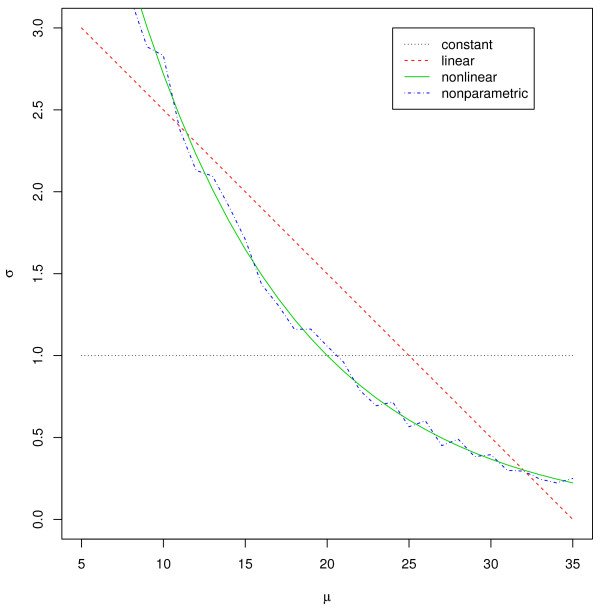
**Confidence intervals of the sensitivities, specificities, and accuracies for duplicate experiments.** File: CI2.pdf - Mean plus or minus one standard error of the sensitivities, specificities, and accuracies of the quantile and projection quantile methods for the simulated data from two experiments (*n *= 2).

Table 1 presents the sensitivities, specificities, and accuracies of the quantile and projection quantile methods for the simulated data from duplicated experiments (*n *= 2) and Figure 4 shows their confidence intervals. The classical methods were not applied because they work only for *n *> 2. Under the constant variance, all the methods performed well. Under the linear, nonlinear, and nonparametric variances, the quantile and projection quantile methods with constant quantile regression performed worse than those with the other quantile regression due to the heterogeneity of the variability, as shown in Cho et al. [4]. When comparing the quantile and projection quantile methods, the latter sometimes had somewhat lower sensitivities than the former. However, the quantile and projection quantile methods are mostly comparable.

**Table 1:** Sensitivities, specificities, and accuracies of the quantile and projection quantile methods for the simulated data from duplicated experiments

		**Simulated Under**			
**n**	**Method**	**Constant**	**Linear**	**Nonlinear**	**Nonparametric**
	Quantile				
	Constant	(85.0, 99.5, 98.8)	(84.7, 93.1, 92.6)	(94.3, 87.6, 87.9)	(94.3, 87.7, 88.0)
	Linear	(85.0, 99.5, 98.8)	(83.7, 99.3, 98.5)	(87.7, 94.7, 94.4)	(87.3, 94.7, 94.3)
	Nonlinear	(85.0, 99.5, 98.8)	(83.3, 99.3, 98.5)	(87.7, 94.8, 94.5)	(86.9, 94.9, 94.5)
	Nonparametric	(79.0, 99.2, 98.2)	(81.6, 99.1, 98.2)	(84.8, 99.0, 98.3)	(84.8, 99.0, 98.3)
2	Projection Quantile				
	Constant	(88.9, 99.1, 98.6)	(69.7, 97.0, 95.7)	(78.6, 94.1, 93.4)	(78.8, 94.1, 93.3)
	Linear	(88.8, 99.1, 98.5)	(86.5, 98.9, 98.3)	(88.5, 96.1, 95.7)	(88.2, 96.1, 95.7)
	Nonlinear	(88.8, 99.1, 98.5)	(86.5, 98.9, 98.3)	(88.3, 98.0, 97.6)	(87.9, 98.0, 97.4)
	Nonparametric	(83.2, 98.7, 97.9)	(84.4, 98.7, 98.0)	(86.6, 98.6, 98.0)	(86.0, 98.5, 97.9)

Table 2 presents the sensitivities, specificities, and accuracies of the classical and projection quantile methods for the simulated data from three to eight experiments (3 ≤* n *≤ 8) and Additional File 1 shows their confidence intervals. The results are not shown for *n *≥ 9. With multiple experiments, the projection quantile methods with constant, linear, nonlinear, and nonparametric quantile regression performed like those with duplicated experiments. When *n* = 3, the classical methods had very low sensitivities, resulting in the lower accuracies. With increasing *n*, the sensitivities of the classical methods increased. When *n* = 7 or 8, Glubbs’ test was comparable to the projection quantile methods with linear, nonlinear, and nonparametric quantile regression. This implies that the classical methods require a sufficiently large number of replicates. In reality, experiments are often repeated three or more times; thus, the projection quantile method is practically very useful.

**Table 2:** Sensitivities, specificities, and accuracies of the classical and projection quantile methods for the simulated data from multiple experiments

					
		**Simulated Under**			
**n**	**Method**	**Constant**	**Linear**	**Nonlinear**	**Nonparametric**
	Classical				
	Dixon	(10.5, 94.9, 90.7)	(17.3, 94.9, 91.0)	(18.5, 94.9, 91.1)	(17.7, 94.9, 91.0)
	Grubbs	(20.8, 89.9, 86.5)	(30.1, 89.9, 87.0)	(34.4, 89.9, 87.2)	(33.7, 90.0, 87.2)
	Projection Quantile				
3	Constant	(90.6, 99.5, 99.0)	(56.0, 98.5, 96.4)	(58.8, 95.7, 93.9)	(57.9, 95.7, 93.8)
	Linear	(90.4, 99.5, 99.0)	(84.0, 99.3, 98.5)	(85.1, 96.5, 95.9)	(84.8, 96.6, 96.0)
	Nonlinear	(90.4, 99.5, 99.0)	(84.0, 99.3, 98.5)	(84.8, 98.5, 97.8)	(83.5, 98.4, 97.7)
	Nonparametric	(85.3, 99.2, 98.5)	(82.0, 99.1, 98.2)	(83.5, 99.0, 98.2)	(83.2, 99.0, 98.2)
	Classical				
	Dixon	(29.7, 95.0, 91.7)	(44.1, 95.0, 92.4)	(54.9, 94.9, 92.9)	(54.5, 94.9, 92.9)
	Grubbs	(49.6, 90.0, 88.0)	(61.1, 90.0, 88.6)	(71.2, 90.0, 89.1)	(70.2, 89.9, 89.0)
	Projection Quantile				
4	Constant	(89.4, 99.6, 99.1)	(46.4, 99.1, 96.5)	(44.3, 97.2, 94.6)	(43.8, 97.3, 94.6)
	Linear	(89.3, 99.6, 99.0)	(86.8, 99.5, 98.8)	(86.3, 97.0, 96.5)	(86.4, 97.2, 96.6)
	Nonlinear	(89.3, 99.6, 99.0)	(86.8, 99.5, 98.8)	(87.5, 99.2, 98.6)	(87.8, 99.1, 98.5)
	Nonparametric	(84.8, 99.3, 98.6)	(84.5, 99.3, 98.5)	(86.5, 99.2, 98.5)	(85.9, 99.1, 98.4)
	Classical				
	Dixon	(51.5, 94.6, 92.4)	(63.0, 94.6, 93.0)	(73.0, 94.6, 93.5)	(72.6, 94.6, 93.5)
	Grubbs	(70.7, 90.0, 89.0)	(77.0, 90.0, 89.4)	(82.3, 90.0, 89.6)	(82.0, 90.1, 89.7)
	Projection Quantile				
5	Constant	(89.2, 99.6, 99.1)	(40.0, 99.5, 96.5)	(35.9, 97.9, 94.8)	(35.0, 97.9, 94.8)
	Linear	(89.0, 99.6, 99.1)	(87.3, 99.5, 98.9)	(85.5, 97.5, 96.9)	(84.6, 97.6, 96.9)
	Nonlinear	(89.0, 99.6, 99.1)	(87.3, 99.5, 98.9)	(87.2, 99.3, 98.7)	(86.2, 99.2, 98.6)
	Nonparametric	(84.1, 99.4, 98.6)	(84.2, 99.3, 98.5)	(86.9, 99.0, 98.4)	(86.0, 99.0, 98.4)
	Classical				
	Dixon	(66.0, 94.4, 92.9)	(73.3, 94.4, 93.3)	(79.6, 94.4, 93.6)	(79.9, 94.5, 93.8)
	Grubbs	(81.1, 90.0, 89.6)	(82.9, 90.0, 89.7)	(86.1, 90.0, 89.8)	(86.0, 90.2, 90.0)
	Projection Quantile				
6	Constant	(87.6, 99.6, 99.0)	(34.1, 99.6, 96.4)	(29.7, 98.2, 94.8)	(29.7, 98.4, 94.9)
	Linear	(87.4, 99.6, 99.0)	(85.9, 99.5, 98.8)	(82.5, 97.9, 97.1)	(82.7, 98.0, 97.2)
	Nonlinear	(87.4, 99.6, 99.0)	(85.9, 99.5, 98.8)	(85.7, 99.3, 98.1)	(85.0, 99.2, 98.5)
	Nonparametric	(82.8, 99.3, 98.5)	(83.4, 99.3, 98.5)	(86.0, 99.2, 98.6)	(85.8, 99.1, 98.5)
	Classical				
	Dixon	(73.2, 94.3, 93.2)	(78.4, 94.3, 93.5)	(83.5, 94.3, 93.7)	(83.6, 94.3, 93.8)
	Grubbs	(85.8, 90.0, 89.8)	(86.5, 90.1, 89.9)	(88.2, 90.1, 90.0)	(88.0, 90.2, 90.0)
	Projection Quantile				
7	Constant	(86.2, 99.6, 99.0)	(30.2, 99.8, 96.3)	(26.3, 98.6, 95.0)	(26.1, 98.6, 95.0)
	Linear	(85.8, 99.6, 98.9)	(85.6, 99.5, 98.8)	(81.4, 98.3, 97.5)	(80.4, 98.3, 97.4)
	Nonlinear	(85.8, 99.6, 98.9)	(85.6, 99.4, 98.7)	(85.9, 99.5, 98.8)	(84.7, 99.3, 98.6)
	Nonparametric	(80.8, 99.3, 98.4)	(82.3, 99.3, 98.5)	(86.2, 99.2, 98.6)	(85.8, 99.2, 98.5)
	Classical				
	Dixon	(71.2, 94.5, 93.4)	(76.7, 94.5, 93.6)	(82.4, 94.5, 93.9)	(82.7, 94.5, 93.9)
	Grubbs	(89.1, 90.0, 90.0)	(87.7, 90.0, 89.9)	(89.2, 90.0, 90.0)	(89.3, 90.0, 89.9)
	Projection Quantile				
8	Constant	(85.9, 99.7, 99.0)	(26.5, 99.8, 96.1)	(23.2, 98.0, 94.2)	(24.1, 97.9, 94.2)
	Linear	(85.7, 99.6, 98.9)	(84.8, 99.4, 98.7)	(77.1, 98.1, 97.0)	(77.3, 98.1, 97.1)
	Nonlin	(85.7, 99.6, 98.9)	(84.8, 98.8, 98.1)	(84.4, 99.4, 98.7)	(84.0, 99.3, 98.5)
	Nonparametric	(80.2, 99.4, 98.4)	(81.6, 99.3, 98.4)	(85.7, 99.2, 98.5)	(86.2, 99.1, 98.5)

### Real-life data

We here illustrate the projection quantile approach with real-life data obtained from three replicates of LC/MS/MS experiments with 922 peptides (*n *= 3 and *p *= 922). The details of the experiments can be found in Min et al. [11] and Cho et al. [4]. Here, the primary goal of the analysis is to detect outliers automatically in the pre-processing step prior to further analysis.

**Figure 5:**
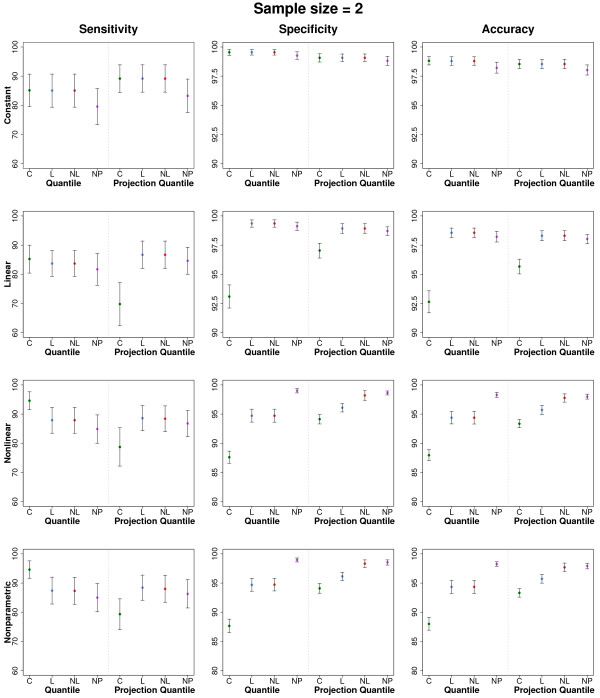
**3-D Scatter plot of LC/MS data with three replicates.** File: Scatter3d.png - Scatter plot of LC/MS data with three replicates; The straight line at the center is the first PC vector. C = Contant, L = Linear, NL = Nonlinear, NP = Nonparametric.

To use the projection approach, we first investigate how much the first PC explains the variation in the data. The first PC takes 96.9% of the variation and the second and third PCs take 1.73% and 1.34%, respectively. This supports that it is enough to use only the first PC. The projection approach with constant, linear, nonlinear, and nonparametric quantile regression selected 74, 69, 99, and 67, respectively. The 3-D scatter plot of the data, shown in Figure 5, revealed the variability of the data to be heterogeneous. Constant quantile regression tended to select more peptides at low levels as outliers, whereas the others selected more peptides at the higher levels.

This implies that the projection approach assuming a constant variance can generate many false positives and/or false negatives and, therefore, that more flexible quantile regression is more appropriate than constant quantile regression.

## Conclusion

We propose an approach for detecting outliers automatically in low replicated, high-throughput data generated from MS experiments. Because of the practical problems such as cost and time, LC/MS data is usually generated by repeating the experiment three or four times under the same technical or biological condition. Outliers can be investigated within each peptide when there are many replicates; however, within-peptide approaches such as Dixon and Grubbs’ tests are crude in the case of few replicates. A quantile regression approach on an MA plot was proposed in Cho et al. [4] when there are only two replicates. Thus, our proposed method can be used when there are two or somewhat more replicates.

The projection approach using various quantile regressions was examined for outlier detection. The projection approach with linear, nonlinear, or nonparametric quantile regression was more appropriate than the others in heterogeneous high-throughput data. The choice among linear, nonlinear, and nonparametric is dependent on the degree of heterogeneity of the data. In addition, our software program provides a number of options. A single method may not be the best in any situation. Therefore, the data can be applied empirically with various options. Moreover, experimental confirmation is needed after applying our automatic outlier detection. Nevertheless, it is useful because manual examination of all observations is time-consuming without pre-screening.

## Availability and Requirements

**Project name:** Outlier Detection for Mass Spectrometry

**Project homepage:**http://statlab.korea.ac.kr/OutlierDM/

**Operating system(s):** Windows, Unix-like systems (Linux, Mac OS X)

**Programming language:** R (the version of R should be ¿ = 2.14.0)

**License:** GNU GPL version 2 or later

## Competing interests

The authors declare that they have no competing interests.

## Author’s contributions

Cho designed and directed this research. Eo wrote and optimized the R code and maintained the software program. Cho and Eo wrote the manuscript. All authors contributed ideas, and read and approved the manuscript.

## Supplementary Material

Additional file 1 Confidence intervals of the sensitivities, specificities, and accuracies for multiple experiments. File: CI3.pdf - Mean plus or minus one standard error of the sensitivities, specificities, and accuracies of the classical and projection quantile methods for the simulated data from multiple experiments (3 ≤* n *≤ 8). Click here for file
