# Supplementary material for: Outlier Detection using Projection Quantile Regression for Mass Spectrometry Data with Low Replication
Source: BMC Res Notes. 2012 May 15;5:236. doi: 10.1186/1756-0500-5-236 (PMC3514222; doi:10.1186/1756-0500-5-236)
Supplement: Additional file 1 — Confidence intervals of the sensitivities, specificities, and accuracies for multiple experiments. File: CI3.pdf - Mean plus or minus one standard error of the sensitivities, specificities, and accuracies of the classical and projection quantile methods for the simulated data from multiple experiments (3 ≤ n ≤ 8). [file 1756-0500-5-236-S1.pdf]

Sample size = 3

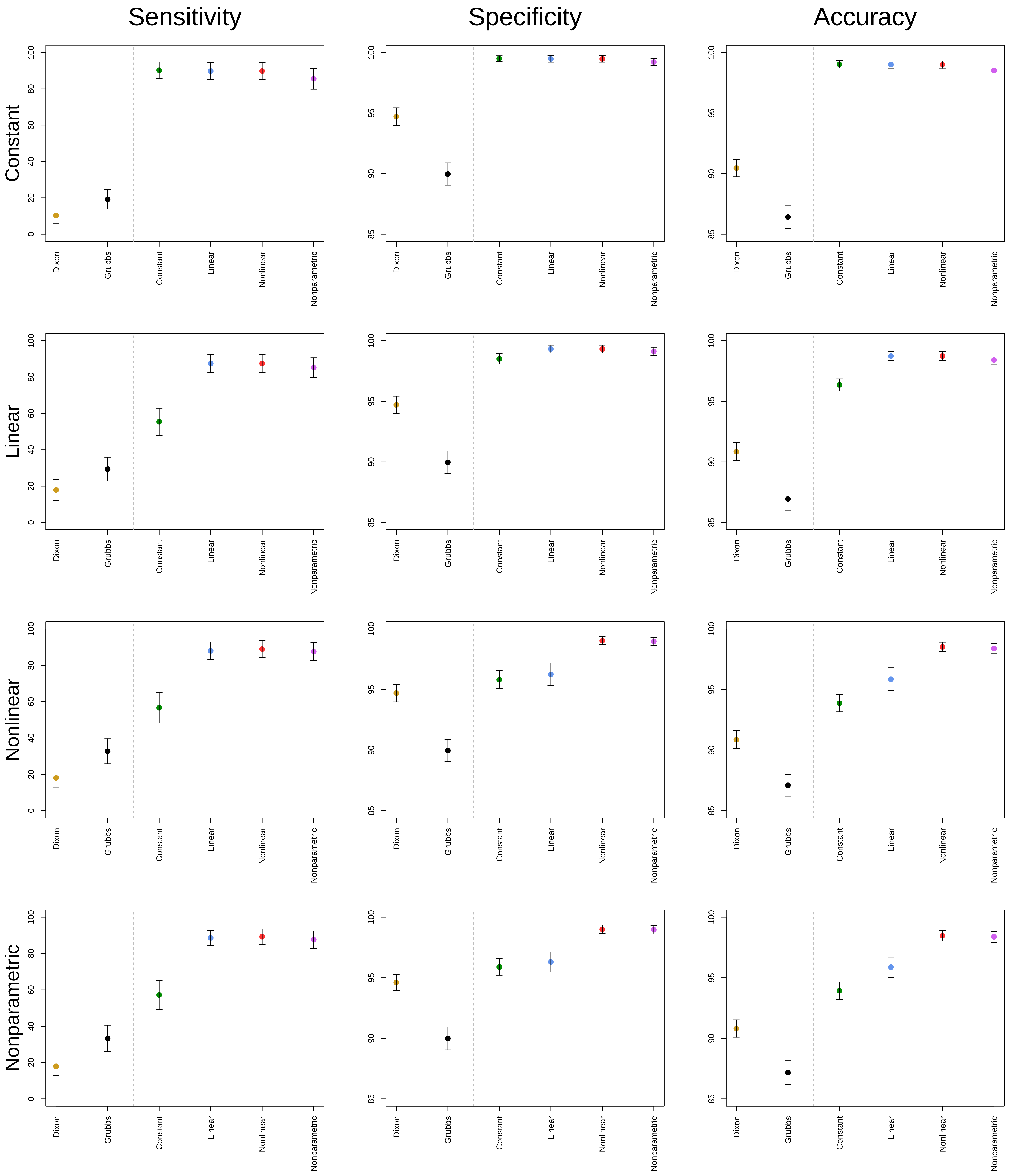

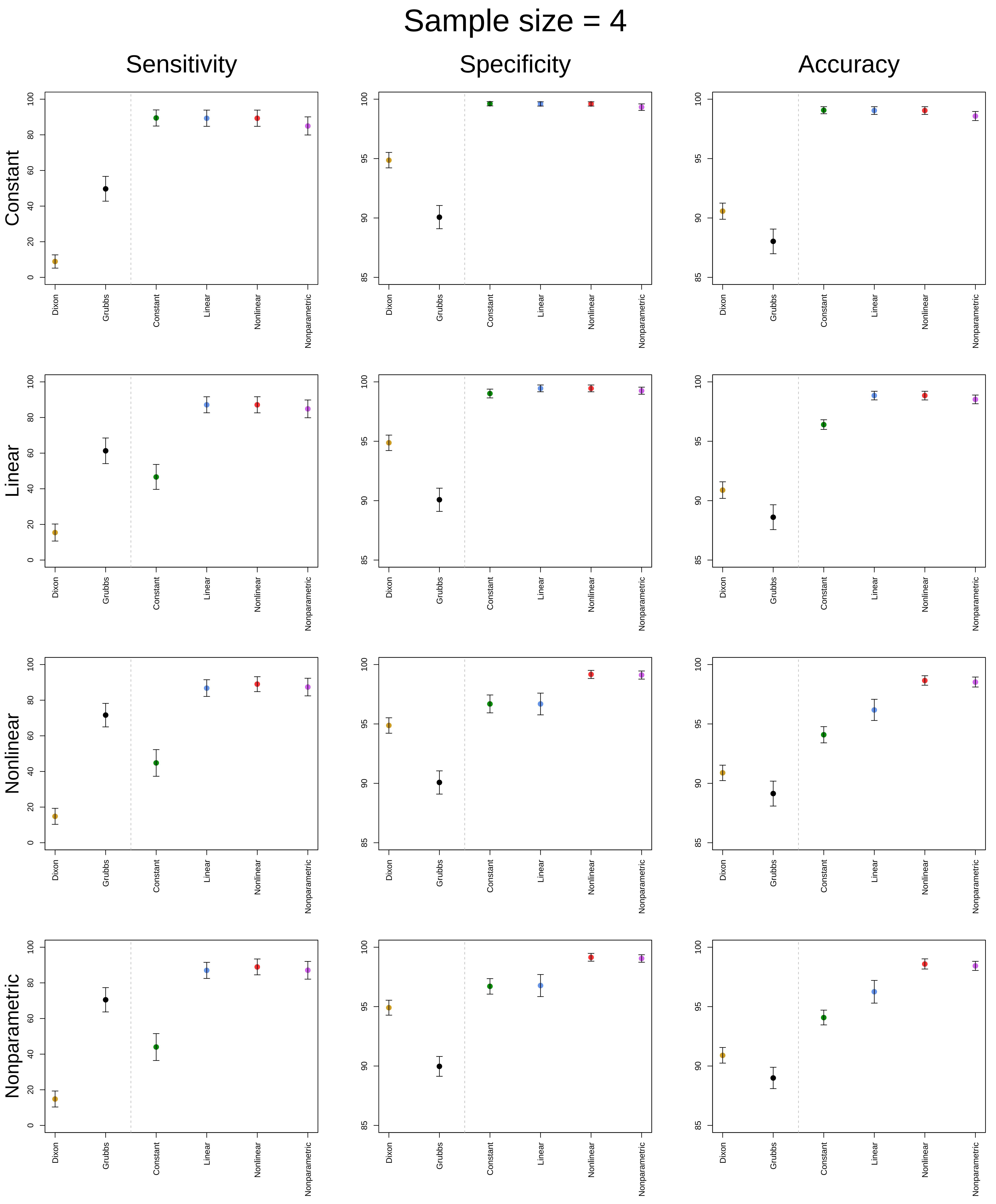

Sample size = 4
Sensitivity
Specificity
Accuracy
Constant
Linear
Nonlinear
Nonparametric
Dixon
Grubbs
Constant
Linear
Nonlinear
Nonparametric

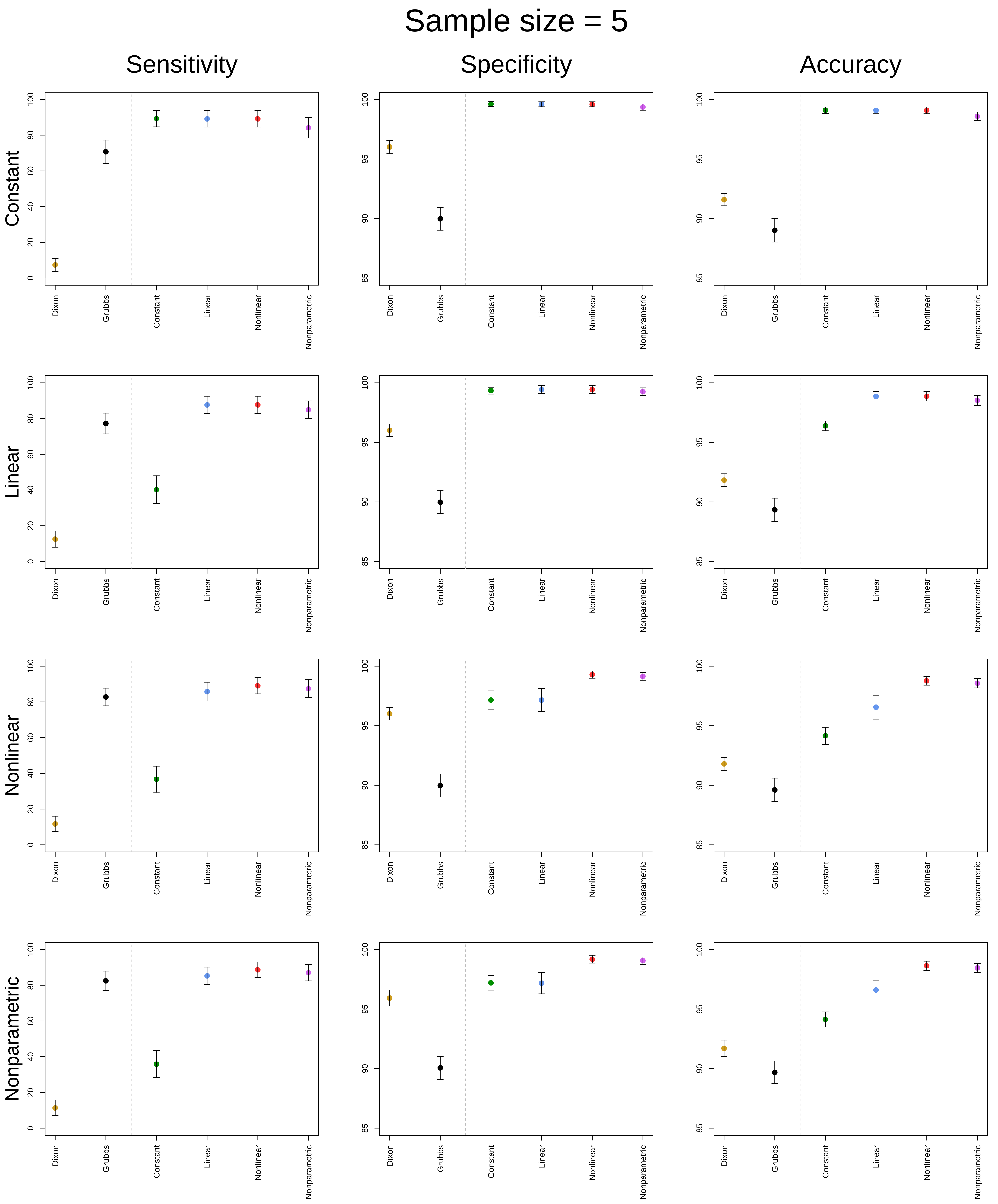
Sample size = 5
Sensitivity
Specificity
Accuracy
Constant
Linear
Nonlinear
Nonparametric
Dixon
Grubbs
Constant
Linear
Nonlinear
Nonparametric

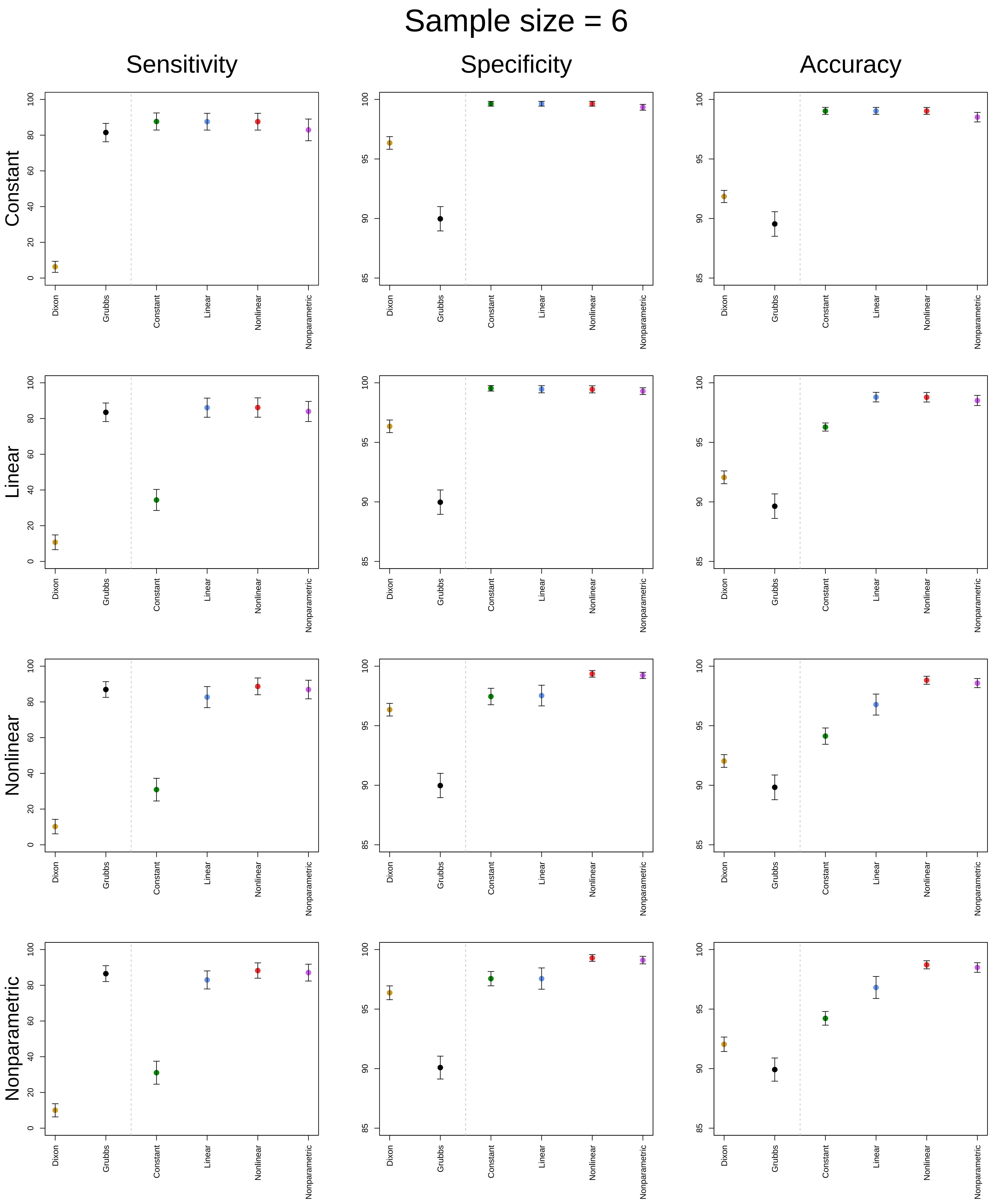
Sample size = 6
Sensitivity
Specificity
Accuracy
Constant
Linear
Nonlinear
Nonparametric
Dixon
Grubbs
Constant
Linear
Nonlinear
Nonparametric

Sample size = 7

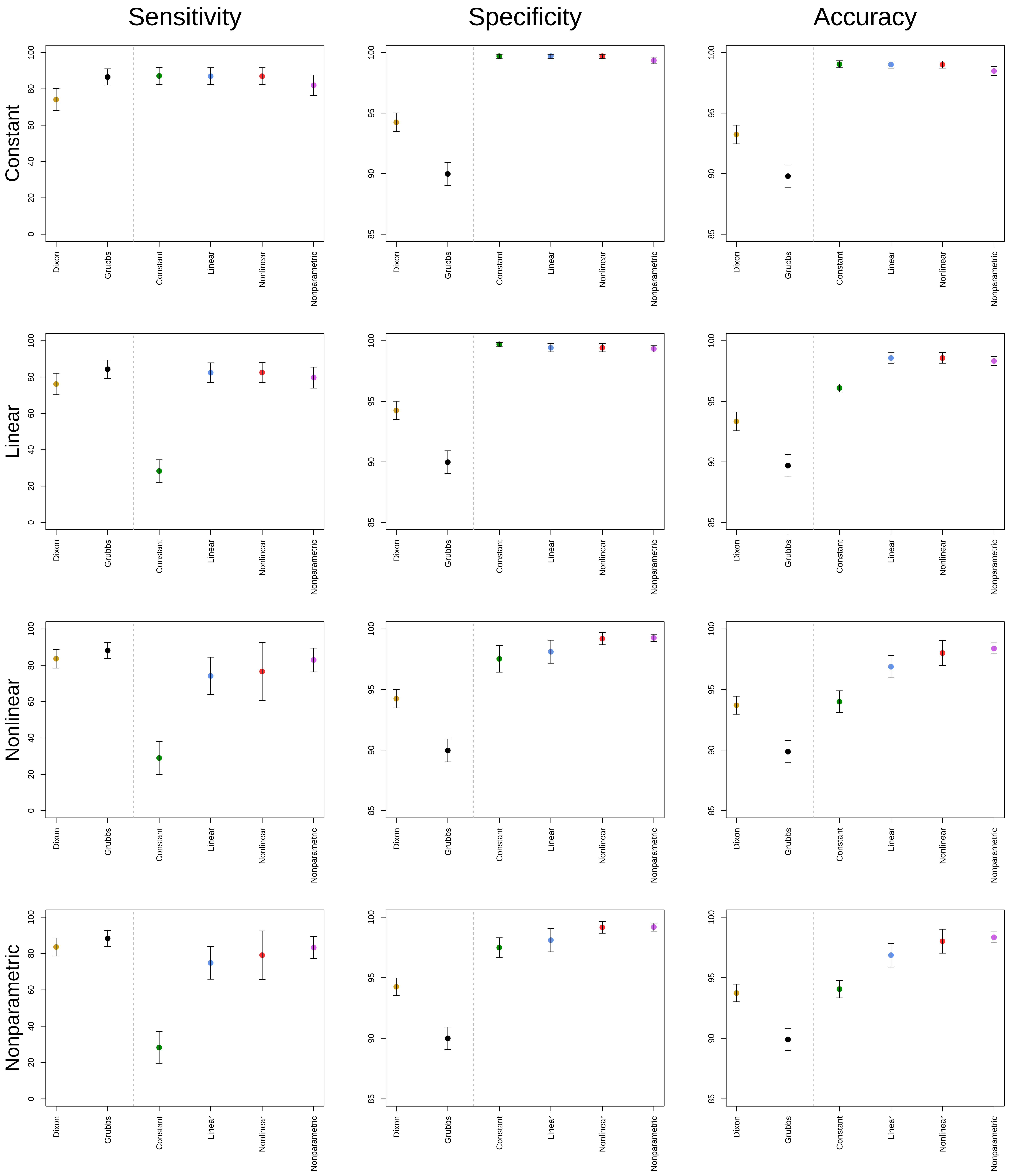

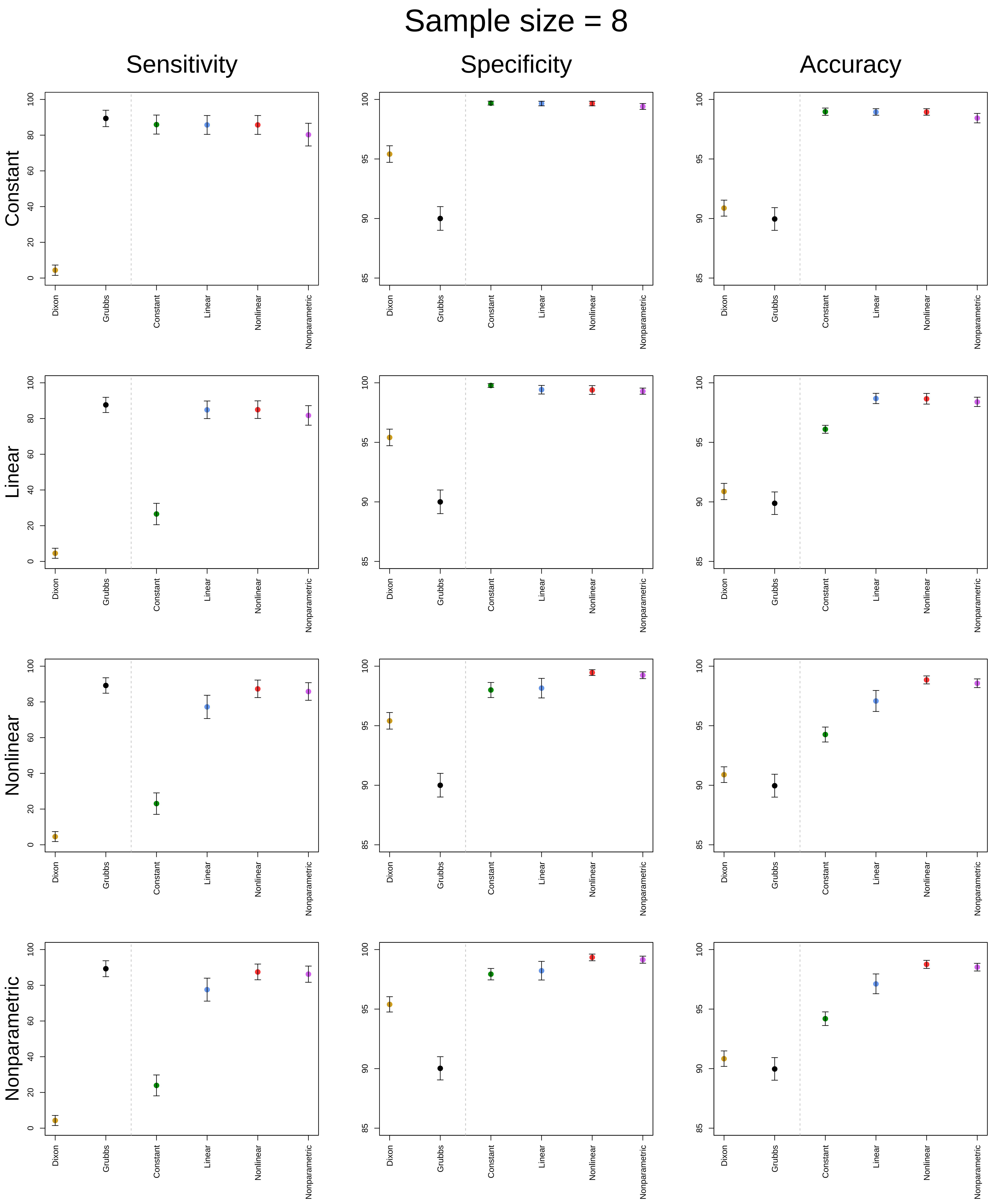

Sample size = 8
Sensitivity
Specificity
Accuracy
Constant
Linear
Nonlinear
Nonparametric
Dixon
Grubbs
Constant
Linear
Nonlinear
Nonparametric
